# Translation and psychometric evaluation of the Chinese version of the resilience scale for children with cancer

**DOI:** 10.1186/s12955-021-01865-y

**Published:** 2021-10-02

**Authors:** Joyce Oi Kwan Chung, William Ho Cheung Li, Xia Wei, Ankie Tan Cheung, Laurie Long Kwan Ho, Godfrey Chi-Fung Chan

**Affiliations:** 1 School of Nursing, The Hong Kong Polytechnic University, Hong Kong, Hong Kong; 2 School of Nursing, The University of Hong Kong, 4/F, William M. W. Mong Block, 21 Sassoon Road, Pokfulam, Hong Kong, Hong Kong; 3 School of Nursing, Sun Yat-Sen University, Guangzhou, China; 4 Department of Adolescent Medicine, Queen Mary Hospital, Hong Kong, Hong Kong

**Keywords:** Child, Neoplasms, Chinese, Psychometrics, Resilience

## Abstract

**Background:**

To test the psychometric properties of a traditional Chinese version of the Resilience Scale for Children (RS-10) and examine its factorial structure via a confirmatory factor analysis (CFA).

**Methods:**

One hundred and eighty-six Hong Kong Chinese children with cancer were recruited in the paediatric oncology units of two public acute-care hospitals in Hong Kong to participate in this cross-sectional study. The psychometric properties of the traditional Chinese version of the RS-10 were assessed, namely its content equivalence, convergent and discriminant validity, construct validity, internal consistency and test–retest reliability.

**Results:**

The newly translated traditional Chinese version of the RS-10 demonstrated adequate internal consistency (Cronbach’s α = .83, McDonald’s Ω = .80), excellent test–retest reliability (.89), good content equivalence (CVI = 96%) and appropriate convergent (r =  − .52, P = .01) and discriminant validity (r = .61, P = .01). The CFA results demonstrated that there was a good fit between the factor structure of the Chinese version of the RS-10 and the observed data (χ^2^/df = 2.34, TLI = .951, RMSEA = .053, CFI = .962, GFI = .948, SRMR = .052), thereby confirming the construct validity of this instrument.

**Conclusions:**

The traditional Chinese version of the RS-10 was found to be a reliable and valid tool for assessing the resilience of Hong Kong Chinese children with cancer. The newly developed traditional Chinese version of the RS-10 is an appropriate clinical research tool for evaluating the effectiveness of nursing interventions in enhancing the resilience of and promoting mental well-being in children with cancer.

*Trial registration* NCT03544190.

## Background

It is well documented that cancer and its treatment have adverse effects on the physical and psychological well-being of children [1–3]. The cumulative physical effects of chemotherapy and/or radiotherapy include damage to normal body tissues and impaired physical fitness, leading to reduced cardiorespiratory function and decreased muscle strength [4]. Such adverse effects may begin at diagnosis and continue for months or years after the completion of therapy [5]. Adverse long-term effects on the psychological well-being of children with cancer include decreased self-esteem and increased anxiety and depression, which can decrease the children’s quality of life [3, 6, 7]. A study on the effects of cancer on Hong Kong Chinese children’s physical, emotional, and psychosocial well-being indicated that children had relatively high anxiety scores at their admission for cancer treatment and that nearly all children hospitalised with cancer expressed varying degrees of sadness and worry [2]. In addition, more than half of the participants were at risk of developing depression or presented some depressive symptoms during their stay in hospital [2].

An increasing number of studies have examined patients’ resilience and adaptation to having cancer [8]. Resilience is defined as the ability of an individual to utilise a collection of protective factors, such as personal and social resources and a perceived level of family cohesion, to maintain mental well-being in the face of stress and adversity [9, 10]. Numerous studies have indicated that resilience prevents the development of mental health problems and is associated with positive mental health outcomes in children and adolescents, such as reduced levels of anxiety, depression and obsessive–compulsive symptoms [11–13]. The assessment of resilience in children with cancer is therefore crucial, as it enables a thorough understanding of their responses to stress and adversity. This understanding is a prerequisite for the design of appropriate psychological interventions to enhance such children’s resilience and foster the development of their coping mechanisms and positive mental well-being. Previous studies, conducted in Western countries, have examined the relationships between resilience and psychological well-being in adult patients with cancer [8, 14, 15]. The results of these studies revealed that resilience is an important psychological predictor of quality of life and that higher resilience in cancer patients was associated with better psychological well-being. However, there is a paucity of research on resilience in children with cancer; most studies have focused on promoting resilience in parents, caregivers or other family members of children with cancer [16, 17]. A review of the literature revealed that no study has examined resilience in children with cancer and how it affects their psychological well-being in the Hong Kong Chinese context. This is a notable research gap, as culture appears to play a critical role in the expression of resilience by children and adolescents [18]. Additionally, as there is vastly different in cultural context between Hong Kong and from Western countries, the way in which Chinese children view the nature and meaning of their cancer may different to their counterparts. Consequently, the resilience and adaptation to diagnosis and treatment of Chinese children may differ considerably from that of Western children. Hence, cultural adaptation of existing resilience-assessment tools should be conducted before it can be used to measure resilience for Hong Kong Chinese children with cancer.

One scale for assessing resilience is the resilience scale (RS). The RS is a 25-item scale that uses a 7-point rating and was developed by Wagnild and Young based on a conceptual model derived from a qualitative study of a group of women who exhibited adaptation after a major life event [19]. The five characteristics that serve as the conceptual foundation of resilience are a sense of meaning and purpose, authenticity, equanimity, self-reliance and perseverance. The psychometric properties of the RS were tested and validated, and the results demonstrated its adequate internal consistency reliability [19]. The concurrent validity of the RS was supported by the identification of significant correlations between the RS scores and the measures of morale, life satisfaction and depression [19]. A factor analysis indicated that the RS assesses two factors: personal competence and the acceptance of self and life [17]. A review of the RS confirmed that its internal consistency was consistently high and its construct validity was appropriate [20]. The RS has been translated into and validated in a variety of languages, and it has been widely used by researchers and healthcare professionals to measure resilience in adolescents and adults in various populations over the past decade [20].

Subsequent to the validation of the RS, the Resilience Scale for Children (RS-10) was developed to measure children’s capacity to respond to life changes [21]. The RS-10 is positively worded and easily understood by children as young as 7 years old and has been translated from English into Arabic and Swedish [21]. However, it has not been used to assess resilience in Hong Kong Chinese children. Given that resilience is a personality characteristic that moderates the response to stress and enhances the ability to face adversity, and thereby promotes adaptation [9, 10], it is of paramount importance that the resilience of Hong Kong Chinese children with cancer is assessed and understood to enable the development of appropriate interventions for them. It is vital therefore to first translate the RS-10 to Chinese. Moreover, it is crucial to evaluate both its linguistic and cultural equivalence. Additionally, the psychometric properties of the Chinese version of the RS-10 needed to be empirically tested.

This study aimed to translate the original English version of the RS-10 into traditional Chinese. The psychometric properties of the resulting traditional Chinese version of the RS-10 were examined, and its factorial structure was examined using a confirmatory factor analysis (CFA).

## Methods

### Design and setting

A descriptive study was conducted, including translation, cultural adaptation, and validation. Psychometrics of the RS-10 was evaluated through a cross-sectional survey in the paediatric oncology units of Queen Mary Hospital and Hong Kong Children’s Hospital in Hong Kong.

### Participants

To be eligible for this study, children were required (1) to be aged between 7 and 14 years; (2) to have been diagnosed with cancer within the previous 6 months and to be currently undergoing active treatments, and (3) to be able to speak Cantonese and read Chinese. As children younger than 7 years may have limited verbal and cognitive capacities to express themselves and may be perplexed by the questionnaire items, only children aged 7–14 years were invited to participate in the study. Children with cognitive and learning problems, who were identified from the medical records, were excluded.

There are no clear rules on the sample size that is required for a factor analysis, and there is no accepted minimal sample criterion. However, the COSMIN guidelines suggests that seven subjects per item and ≥ 100 is very good for factor analysis, but a large sample is better, especially when the data are not normally distributed [22, 23]. In addition, Gorsuch recommends a minimum of 200 subjects for a CFA [24]. Thus, we originally planned to recruit a convenience sample of 200 subjects for testing the psychometric properties of the traditional Chinese version of the RS-10 during the study period. However, the data collection was suspended due to the relocation of services from Queen Mary Hospital to Hong Kong Children’s Hospital from 27 May 2019 to 31 August 2019. Moreover, the social unrest from mid-November to mid-December 2019 affected the progress of subject recruitment. Furthermore, the coronavirus disease 2019 (COVID-19) pandemic meant that the research assistant could not go to the hospital to collect data till the end of the data collection period.

Thus, during the COVID-19 pandemic, we invited a paediatric oncology nurse working in the Hong Kong Children’s Hospital to help to collect the data after her normal duties were finished. We also applied for an extension of the data collection period from 31 March 2020 to 31 December 2020. Nevertheless, the above-mentioned difficulties that we encountered meant that we were able to recruit only 186 subjects (93% of the planned sample size) during the data collection period, which met the requirement of at least seven subjects per item and ≥ 100 in this study [23]. A simulation study shows that a sample size of 150 is reasonable for a simple CFA model [25].We therefore consulted a biostatistician working in a university, who reassured us that a sample size of 186 was sufficient to test the psychometric properties of the traditional Chinese version of the RS-10 and to perform a CFA. We therefore decided not to apply for a further extension of the data collection period and ceased data collection as scheduled on 31 December 2020.

### Measures

#### Resilience scale for children (RS10)

The RS10 is constructed at a 2.4 Flesch–Kincaid reading level [21], which facilitates comprehension and is appropriate for children aged 7 and above. It is a 10-item scale, with each item evaluated on a 4-point Likert scale (1 = ‘not at all like me’, 2 = ‘not much like me’, 3 = ‘somewhat like me’ and 4 = ‘a lot like me’); total possible scores thus range from 10 to 40, with higher scores indicating higher levels of resilience.

#### Center for epidemiologic studies depression scale for children (CES-DC)

The CES-DC comprises 20 standardised items to evaluate depressive symptoms. All items are evaluated on a 4-point Likert scale (0 = ‘not at all’, 1 = ‘a little’, 2 = ‘some’, 3 = ‘a lot’) in relation to their incidence during the previous week; total possible scores thus range from 0 to 60, with higher scores indicating more depressive symptoms. The psychometric properties of the Chinese version of the CES-DC have been empirically tested, and it was found to have an internal consistency coefficient (Cronbach’s α) of 0.82, a convergent construct validity r = 0.63 (*p* < 0.01) and a discriminant construct validity r =  − 0.52 (*p* < 0.01) [26].

#### Rosenberg's self-esteem scale (RSES)

The RSES was designed to measure self-esteem as a global disposition and has been widely used to assess children [26]. The RSES consists of 10 items rated on a 4-point Likert scale ranging from 1 (‘strongly disagree’) to 4 (‘strongly agree’), with total possible scores ranging from 10 to 40. Higher scores indicate higher levels of self-esteem. The Chinese version of the RSES has been used to assess children and was found to have a Cronbach’s α of 0.84 and a discriminant construct validity of r =  − 0.52 (*p* < 0.01) [26].

### Data collection

Ethical approval for the study was obtained from the Institutional Review Board of the University of Hong Kong/Hospital Authority Hong Kong West Cluster and the Hong Kong Children’s Hospital. Written consent was obtained from children’s parents after they were informed of the purpose of the study. Parents were given the option of allowing or refusing their child’s involvement in the study, and children were informed that their participation was voluntary and were invited to put their names on a special assent form. Children were then asked to respond to the printed questionnaires, including RS10, CES-DC, and RSES.

#### Process of translation of the RS-10

The RS-10 was translated and back-translated according to the World Health Organization guidelines on the process of translation and adaptation of instruments [27]. Thus, the 10 items of the RS-10 were first translated from English to traditional Chinese by a paediatric nurse specialist who is familiar with the concept of resilience in children (forward translation). Subsequently, a second translator, who was blinded to original items, performed the back-translation. The preservation of conceptual rather than literal meaning was the aim of the translation. The retranslated English version and the original English version were then compared to confirm that the meaning of each item had been maintained. Discrepancies were resolved by the nurse specialist and the back-translator via discussion.

### Statistical analysis

#### Semantic and content equivalence

A panel of experts was set up to test the semantic and content equivalence of the traditional Chinese version of the RS-10. The panel comprised a paediatric oncology nurse specialist, a paediatric oncology researcher, and an assistant professor and an associate professor who work at a local university. All of the panel members were bilingual and had experience of translating and validating instruments. The panel was asked to rate the translation equivalence of each item on a 4-point scale (from 1 = ‘not equivalent’ to 4 = ‘most equivalent’). An equivalence rate was then calculated for each item, and any item that was considered not equivalent, by being rated 1 or 2 by more than 20% of respondents, was amended. To assess the content equivalence, the panel was asked to rate each item on a 4-point scale (from 1 = ‘not relevant’ to 4 = ‘very relevant’). The content validity index (CVI) was used to assess the average content equivalence of all individual items on the scale that were rated as either 3 or 4. A CVI score of 90% or above is generally considered as an indication of good content equivalence [28].

The scale scores were summarized using descriptive statistics, with the floor and ceiling percentages, and the distribution of the responses reported to assess the scaling properties of the questionnaire. The floor and ceiling percentages of a scale indicate the percentages of participants with the lowest and highest plausible scale scores, respectively.

#### Construct validity testing

Convergent validity was established by determining the correlations between scores on the Chinese versions of the RS-10 and the RSES using the Pearson product–moment correlation coefficient. Our previous study indicated that Chinese adolescents with greater resilience have higher self-esteem [29]. We hypothesised that there would be a positive correlation between scores on the traditional Chinese version of the RS-10 and scores on the Chinese version of the RSES. Discriminant validity was estimated by examining the correlation between scores on the RS-10 and the CES-DC.

Factorial validity was evaluated by a CFA. The Tucker–Lewis index (TLI), the root-mean-square error of approximation (RMSEA), the comparative fit index (CFI), the goodness-of-fit (GFI) and the standardised root-mean-square residual (SRMR) were used to evaluate the goodness of fit of the factor analysis models. Cut-off values of ≥ 0.95, ≤ 0.06, ≥ 0.95, ≥ 0.90 and ≤ 0.08 were used for the TLI, the RMSEA, the CFI, the GEI and the SRMR, respectively [30, 31]. The diagonally weighted least squares (DWLS) estimator was used to assess the ordinal variables in the RS-10, where values of 0.32, 0.45, 0.55, 0.63 and 0.71 indicate poor, fair, good, very good and excellent factor loadings, respectively [32]. Items with factor loadings of < 0.40 were removed. Initial one-factor and two-factor model analyses were then performed using parameters based on the theoretical structure of the instrument and the modification indices of Analysis of Moment Structures software [33]. Finally, the one-factor and two-factor models were modified, and the analyses were re-performed.

#### Reliability testing

The reliability of the traditional Chinese version of the RS-10 was assessed by determining its internal consistency by calculating Cronbach’s α and McDonald's Ω. To examine the stability of the RS-10, all participants were asked to respond to the RS-10 again after 2 weeks, via telephone follow-up. The intraclass correlation coefficient (ICC-consistency) was performed using Two-Way Mixed Model to estimate the test–retest reliability coefficient.

Data analysis above was conducted in IBM SPSS statistics (Version 25) and IBM SPSS Amos (version 23). The McDonald's Ω was calculated using the SPSSS omega extension.

## Results

### Demographics

Table 1 presents the demographic and clinical characteristics of the participants. One hundred boys and 86 girls were recruited for this study, and had a mean age of 10.4 ± 2.5 years. Twenty participants (10.8%) were from single-parent families. Most participants had a diagnosis of leukaemia (46.2%) or brain tumour (23.1%). More than half (58.6%) of the participants received chemotherapy only, and approximately 36.5% received more than one cancer treatment.

**Table 1:** Demographic and clinical characteristics of the participants (N = 186)

	Frequency	%
Sex		
Male	100	53.8
Female	86	46.2
Diagnosis		
Leukaemia	86	46.2
Lymphoma	36	19.4
Brain tumour	43	23.1
Osteosarcomas	17	9.1
Kidney tumour	4	2.2
Treatment received		
Surgery	5	2.7
Radiotherapy	4	2.2
Chemotherapy	109	58.6
Multiple treatments	68	36.5
Parental marital status		
Live with both parents	166	89.2
Single-parent family	20	10.8
Parents’ educational attainment		
Primary school or below	6	3.2
Lower secondary school	46	24.7
Upper secondary school	61	32.8
Tertiary education	73	39.3
Religion		
Yes	54	29.0
No	132	71.0
	Mean	SD
Range of ages (7–14 years)	10.4	2.5

Despite the high distribution of responses “agree” and “strongly agree”, the overall score had no floor and ceiling prevalence (Table 2).

**Table 2:** Summary of RS-10 scores (n = 186)

Scales (no. of items)	n	Mean (SD)	Min	Max	Floor (%)	Ceiling (%)	Distribution of responses (%)			
							SD	D	A	SA
*Personal competence (6)*	182	13.07(1.91)	7	18	0	0				
I finish what I begin	185	3.08(0.60)	1	4			2.2	7.6	70.3	20.0
When I get upset, I know how to calm down	186	2.91(0.64)	1	4			2.2	18.8	64.5	14.5
I like to think about all the things I want to do	184	3.25(0.70)	1	4			2.7	6.5	53.8	37.0
I don't like to give up, even when something is hard to do	185	3.10(0.64)	1	4			0.5	14.1	60.5	24.9
I like to practice hard to get good at what I'm doing	186	3.17(0.54)	2	4			0	7.5	68.3	24.2
When I do something, I want to do it well	186	3.37(0.55)	2	4			0	3.2	57.0	39.8
*Acceptance of self and life (4)*	186	12.25(2.03)	6	16	0	9.7				
I am excited to learn new things	186	3.14(0.64)	2	4			0	14.5	57.0	28.5
I am happy with myself	186	3.24(0.67)	1	4			1.6	8.1	54.8	35.5
I like to find something to laugh or smile about everyday	186	3.02(0.70)	1	4			1.1	19.9	54.8	24.2
I think I'm okay just the way I am right now	186	2.84(0.72)	1	4			1.6	30.1	50.5	17.7
*Overall score (10)*	182	25.33(3.44)	16	34	0	0				

*SD* strongly disagree, *D* disagree, *A* Agree, *SA* strongly agree

### Validity

#### Semantic equivalence

The semantic equivalence of the items in the traditional Chinese version of the RS-10 ranged from 86 to 100%. This demonstrated that the meanings of the items in the translated version were equivalent to those of the items in the original version.

#### Content equivalence

The CVI was 96%, indicating that the content of the traditional Chinese version of the RS-10 was valid.

#### Construct validity

The Pearson product-moment correlation was used to examine the intercorrelations between the scores on the traditional Chinese versions of the RS-10, the CES-DC and the RSES. Small, medium and large correlations are generally represented by correlation coefficients of 0.10– 0.29, 0.30– 0.49 and 0.50 –1.0, respectively [34]. The results showed that there was a strong negative correlation (r =  − 0.52, n = 186 and P = 0.01) between the traditional Chinese versions of the RS-10 and the CES-DC, indicating that greater resilience in children was associated with fewer self-reported depressive symptoms. A strong positive correlation (r = 0.61, n = 186, P = 0.01) was found between the traditional Chinese versions of the RS-10 and the RSES, demonstrating that the children with greater resilience also reported higher levels of self-esteem. These findings supported both the convergent and divergent validity of the traditional Chinese version of the RS-10.

#### Confirmatory factor analysis (CFA)

**Fig. 1:**
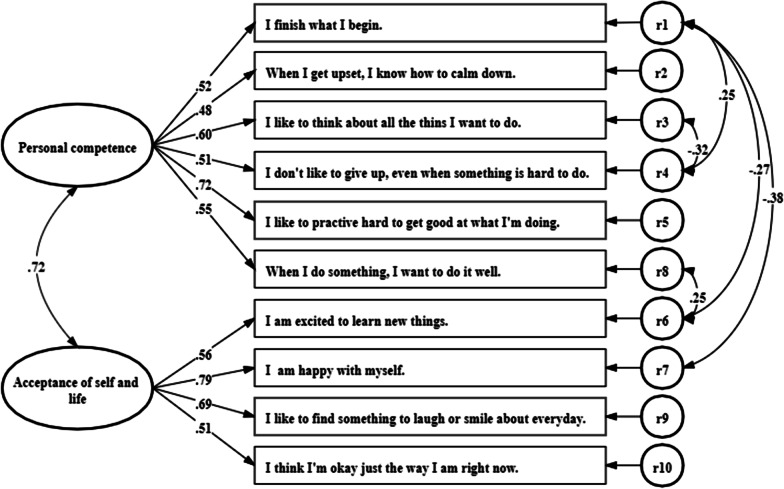
Confirmatory factor analysis for the 2-factor structure model of the Chinese version of RS-10

To evaluate the factorial structure of the traditional Chinese version of the RS-10, a CFA was conducted. A two-factor model was proposed according to the findings of Wagnild and Young [19]. The CFA showed that neither of the initial one-factor and two-factor models had a satisfactory fit. The highest modification index was observed for five pairs of items: r1/r4 (0.25), r1/r6 (− 0.27), r1/r7 (− 0.38), r3/r4 (− 0.32) and r8/r6 (0.25). These pairs of items were modified to have correlated errors. Table 3 summarises the fit indices of the examined CFA models. The modified two-factor model performed well across all fit indices, as the chi-square divided by the degrees of freedom = 2.34, the TLI = 0.951, the RMSEA = 0.053, the CFI = 0.962, the GFI = 0.948 and the SRMR = 0.052. All of the factor loadings were > 0.40, and no items were removed. The items contributing to factor one had factor loadings ranging from 0.48 to 0.72 (i.e., fair to excellent). Items contributing to factor two had factor loadings ranging from 0.51 to 0.79 (i.e., fair to excellent). In addition, the second-order factor loadings of factor one and factor two on the overall latent factor of the scale were excellent, with a value of 0.72. Figure 1 presents the standardised coefficients of the modified two-factor CFA.

**Table 3:** Fit statistics for the factor structure models of the Chinese version of RS-10

Model	χ^2^	df	χ^2^/df	TLI	RMSEA	CFI	GFI	SRMR
*One-factor*								
Initial	179.793	35	5.137	0.622	0.151	0.706	0.834	0.096
Modified*	83.042	29	2.864	0.830	0.101	0.890	0.920	0.070
*Two-factor*								
Initial	136.668	34	4.02	0.724	0.129	0.792	0.876	0.085
Modified*	62.443	28	2.34	0.951	0.053	0.962	0.948	0.052
*Cut-off values*				≥ 0.95	≤ 0.06	≥ 0.95	≥ 0.90	≤ 0.08

*TLI* Tucker–Lewis index, *RMSEA* root mean square error of approximation, *CFI* comparative fit index, *GFI* goodness-of-fit index, *SRMR* standardized root mean square residual

^*^The pairs of items, including r1/r4, r1/r6, r1/r7, r3/r4 and r8/r6 were modified to have correlated errors

### Reliability

The ICC of the traditional Chinese version of the RS-10 at 2-week intervals was 0.89, indicating that the instrument was stable in the population of children with cancer [35]. The internal consistency of the traditional Chinese version of the RS-10 (10 items) was confirmed by a Cronbach’s α of 0.83 (Personal competence: 0.73, Acceptance of self and life: 0.75) and a McDonald’s Ω of 0.80 (Personal competence: 0.73, Acceptance of self and life: 0.75). The corrected item-total correlation coefficients ranged from 0.38 to 0.61. All of the items correlated with the total score on the scale.

## Discussion

The treatment of cancer has been described as an extremely stressful and frightening experience in the life of a child [3, 6, 7]. Taking care of children with cancer therefore presents a major challenge to healthcare professionals, and it is crucial that such professionals develop and evaluate psychological interventions that can help ease the burden of cancer treatment for children. These interventions must also provide support for children to fight cancer and its subsequent adverse treatment effects at every step of their long and difficult journey. A reliable and valid instrument that accurately measures resilience in children with cancer is crucial to enable such interventions to be developed and evaluated. However, no such instrument is available for the assessment of Hong Kong Chinese children with cancer. This study has addressed this research gap by creating and assessing the psychometric properties of a traditional Chinese version of the RS-10 in Hong Kong Chinese children with cancer and examining its factorial structure via a CFA.

The overall results showed that the traditional Chinese version of the RS-10 demonstrated good internal consistency and test–retest reliability, good semantic and content equivalence, and appropriate convergent and discriminant validity. The CFA supported the two-factor structure of the traditional Chinese version of the RS-10. The results suggested that this instrument is appropriate for clinical and research purposes.

The internal consistency of the traditional Chinese version of the RS-10 was high (0.83). This confirms its suitability for research, as a reliability of 0.70 is considered acceptable and a value greater than 0.8 is preferable for an instrument to be used in research [35]. The item–total correlation coefficients indicated that all items were highly correlated with the total scores. The findings suggested that these items are relatively homogeneous and measure the same psychological construct, and are empirical evidence of the reliability of the traditional Chinese version of the RS-10. Even the mode of administration for the test–retest reliability was different, the estimation by the ICC-consistency was high (0.89), indicating that it has good stability for measuring resilience in children with cancer.

The finding of this study also demonstrated the construct validity of the traditional Chinese version of the RS-10. Our results revealed that there was a strong positive correlation between scores on the Chinese version of the RS-10 and scores on the traditional Chinese version of the RSES, indicating that the former scale has construct validity.

There is evidence that resilience is negatively related to depressive symptoms [12, 29, 36]. We hypothesised that there would be a negative correlation between the traditional Chinese version Chinese versions of the RS-10 and the CES-DC. Our results revealed a strong, negative correlation between scores on the traditional Chinese versions of the RS-10 and the CES-DC. This result provided additional evidence that the traditional Chinese version of the RS-10 had construct validity.

The results of a CFA confirmed the factorial structure of the scale. The analysis revealed that the goodness-of-fit indices supported the proposed factor model and demonstrated the congruence between the structures of the traditional Chinese version of the RS-10 and the original English version.

### Implications for research and clinical practice

The findings of this study have important implications for research and clinical practice. The traditional Chinese version of the RS-10 can be adopted by healthcare professionals and the community to assess and monitor the levels of resilience in Hong Kong Chinese children with cancer and those who have survived cancer.

### Limitations

One limitation is that only children with cancer were recruited. It is therefore not clear whether the traditional Chinese version of the RS-10 can differentiate between children with different characteristics. It would be interesting in the future to examine whether there is any difference in resilience between healthy children and those with cancer. Another limitation is that due to the relocation of services from Queen Mary Hospital to Hong Kong Children’s Hospital, the Hong Kong protests and the COVID-19 pandemic, only 186 subjects (93% of the planned sample size) were recruited, despite extending the data collection period. In addition, this study did not evaluate the discriminant validity other than CES-DC and RSES. Future studies may include more constructed-related measures to further evaluate the concurrent validity of the RS-10. Finally, this study targeted to translate and culturally adapt the tools based on the original English scale. The content validity of the scale for the Hong Kong children with cancer is uncertain as we did not assess the comprehensibility of an instrument by the target population after the translation. Future studies should be conducted to determine the content validity of the traditional Chinese version of the RS-10 from the child's perspective.

## Conclusions

This study addressed a key research gap by finding empirical evidence for the Chinese version of RS-10 as a reliable and valid instrument to assess levels of resilience of Hong Kong Chinese children with cancer. The finding of the study also showed consistency between the Chinese version of the scale and the original English version.

## Data Availability

The datasets used and/or analyzed during the current study are available from the corresponding author on reasonable request.

## Abbreviations

CES-DC: Center for epidemiologic studies depression scale for children ()
CFA: Confirmatory factor analysis
CFI: Comparative fit index
COVID-19: The coronavirus disease 2019
CVI: Content validity index
DWLS: Diagonally weighted least squares
GFI: Goodness-of-fit
ICC-consistency: Intraclass correlation coefficient
RMSEA: Root-mean-square error of approximation
RS: Resilience scale
RS-10: Resilience scale for children
RSES: Rosenberg's self-esteem scale
TLI: Tucker–Lewis index

## References

[1] Enskar K, Enskar K, von Essen L (2008). Physical problems and psychosocial function in children with cancer. Pediatr Nurs.

[2] Li HCW, Chung OKJ, Chiu SY (2010). The impact of cancer on children's physical, emotional, and psychosocial well-being. Cancer Nurs.

[3] Li HCW, Williams PD, Lopez V, Chung OKJ, Chiu SY (2013). Relationships among therapy-related symptoms, depressive symptoms, and quality of life in Chinese children hospitalized with cancer: an exploratory study. Cancer Nurs.

[4] Langeveld NE, Grootenhuis MA, Voute PA, Haan RJ, Bos CVD (2004). Quality of life, self-esteem and worries in young adult survivors of childhood cancer. Psychooncology.

[5] Braam KI, van Dijk EM, Veening MA, Bierings MB, Merks JH, Grootenhuis MA, Chinapaw MJ, Sinnema G, Takken T, Huisman J, Kaspers GJ, van Dulmen-den BE (2010). Design of the quality of life in motion (QLIM) study: a randomized controlled trial to evaluate the effectiveness and cost-effectiveness of a combined physical exercise and psychosocial training program to improve physical fitness in children with cancer. BMC Cancer.

[6] Essen LV, Enskar K, Kreuger A, Larsson B, Sjoden PO (2000). Self-esteem, depression and anxiety among Swedish children and adolescents on and off cancer treatment. Acta Paediatr.

[7] Zeltzer LK, Recklitis C, Buchbinder D, Zebrack B, Casillas J, Tsao JC, Lu Q, Krull K (2009). Psychological status in childhood cancer survivors: a report from the childhood cancer survivor study. J Clin Oncol.

[8] Hou WK, Law CC, Yin J, Fu YT (2010). Resource loss, resource gain, and psychological resilience and dysfunction following cancer diagnosis: a growth mixture modeling approach. Health Psychol.

[9] Luthar SS, Cicchetti D, Becker B (2000). The construct of resilience: a critical evaluation and guidelines for future work. Child Dev.

[10] Davydov DM, Stewart R, Ritchie K, Chaudieu I (2010). Resilience and mental health. Clin Psychol Rev.

[11] Hjemdal O, Aune T, Reinfjell T, Stiles TC, Friborg O (2007). Resilience as a predictor of depressive symptoms: a correlational study with young adolescent. Clin Child Psychol Psychiatry.

[12] Hjemdal O, Vogel PA, Solem S, Hagen K, Stiles TC (2011). The relationship between resilience and levels of anxiety, depression, and obsessive–compulsive symptoms in adolescents. Clin Psychol Psychother.

[13] Sun J, Stewart DE, Lovat T, Toomey R, Clement N (2010). Promoting student resilience and wellbeing: Asia-Pacific resilient children and communities project. International research handbook on values education and student wellbeing.

[14] Tian J, Hong JS (2013). Validation of the Chinese version of the Resilience Scale and its cutoff score for detecting low resilience in Chinese cancer patients. Support Care Cancer.

[15] Strauss B, Brix C, Fischer S, Leppert K, Füller J, Roehrig B, Schleussner C, Wendt TG (2007). The influence of resilience on fatigue in cancer patients undergoing radiation therapy (RT). J Cancer Res Clin Oncol.

[16] Rosenberg AR, Baker KS, Syrjala KL, Back AL, Wolfe J (2013). Promoting resilience among parents and caregivers of children with cancer. J Palliat Med.

[17] Rosenberg AR, Bradford MC, Junkins CC, Taylor M, Zhou C, Sherr N, Kross E, Curtis JR, Yi-Frazier JP (2019). Effect of the promoting resilience in stress management intervention for parents of children with cancer (PRISM-P): a randomized clinical trial. JAMA Netw Open.

[18] Arrington EG, Wilson MN (2000). A re-examination of risk and resilience during adolescence: incorporating culture and diversity. J Child Fam Stud.

[19] Wagnild GM, Young HM (1993). Development and psychometric evaluation of the Resilience Scale. J Nurs Meas.

[20] Windle G, Bennett KM, Noyes J (2011). A methodological review of resilience measurement scales. Health Qual Life Outcomes.

[21] The Resilience Center. http://www.resiliencecenter.com/assessments/resilience-scale-for-children-rs10/, Accessed 1 Feb 2018.

[22] Hu L, Bentler PM, Kano Y (1992). Can test statistics in covariance structure analysis be trusted?. Psychol Bull.

[23] Mokkink LB, Prinsen C, Patrick DL, et al. COSMIN methodology for systematic reviews of patient-reported outcome measures (PROMs). User Manual. 2018;78(1). 10.1007/s11136-018-1798-3 PMC5891568 29435801

[24] Gorsuch RL (1983). Factor analysis.

[25] Muthén LK, Muthén BO (2002). How to use a monte carlo study to decide on sample size and determine power. Struct Equ Model.

[26] Li HCW, Chung OKJ, Ho KYE (2010). Center for epidemiologic studies depression scale for children: psychometric testing of the chinese version. J Adv Nurs.

[27] World Health Organization (2016). Process of translation and adaptation of instruments.

[28] Waltz CF, Strickland OL, Lenz ER (2005). Measurement in nursing and health research.

[29] Chung JOK, Lam KK, Ho KY, Cheung AT, Ho LLK, Gibson F, Li WHC (2020). Relationships among resilience, self-esteem, and depressive symptoms in Chinese adolescents. J Health Psychol.

[30] Brown TA (2006). Confirmatory factor analysis for applied research.

[31] Mvududu NH, Sink CA (2013). Factor analysis in counseling research and practice. Couns Outcome Res Eval.

[32] Swank JM, Lambie GW, Witta EL (2012). The counseling competencies scale: a measure of counseling skills, dispositions, and behaviors. Couns Educ Superv.

[33] Jackson PR, Wall TD, Martin R, Davids K (1993). New measures of job control, cognitive demand, and production responsibility. J Appl Psychol.

[34] Portney LG, Watkins MP (2009). Foundations of clinical research: applications to practice.

[35] Nunally JC, Bernstein IH (1994). Psychometric theory.

[36] Li M, Wang L (2016). The associations of psychological stress with depressive and anxiety symptoms among Chinese bladder and renal cancer patients: the mediating role of resilience. PLoS ONE.

